# Comparação dos Resultados entre os Fenômenos de No-Reflow e Slow-Flow Coronariano em Pacientes sem IAMSSST

**DOI:** 10.36660/abc.20190905

**Published:** 2021-05-06

**Authors:** Mustafa Ahmet Huyut

**Affiliations:** 1 Yeni Yuzyil University Faculty of Medicine Department of Cardiology Istambul Turquia Yeni Yuzyil University, Faculty of Medicine, Department of Cardiology, Istambul – Turquia.

**Keywords:** Infarto do Miocárdio, Síndrome Coronariana Aguda, Fenômeno de Não Refluxo, Intervenção Coronária Percutânea/complicações, Fatores de Risco, Angiografia Coronária, Acidente Vascular Cerebral

## Abstract

**Fundamento:**

Os fenômenos de *slow-flow* (CSFP) e *no-reflow* coronariano (CNP) estão associados a um risco aumentado de eventos cardiovasculares adversos maiores (ECAM).

**Objetivos:**

Este estudo teve como objetivo avaliar e comparar os resultados do seguimento clínico de um ano entre pacientes com CNP e CSFP submetidos a intervenções coronárias percutâneas (ICP) em infarto agudo do miocárdio sem supradesnivelamento do segmento ST (IAMSSST).

**Métodos:**

Este estudo incluiu um total de 858 pacientes com diagnóstico de IAMSSST e submetidos a ICP nas 24 horas desde o início dos sintomas. Os pacientes foram divididos em dois grupos, o grupo CSFP (n = 221) e o grupo CNP (n = 25), considerando as características angiográficas do fluxo da trombólise no infarto do miocárdio (TIMI) e na artéria relacionada ao infarto. Os pacientes tiveram um seguimento de um ano. Um valor de p <0,05 foi considerado significativo.

**Resultados:**

O CNP foi observado em 2,91% e o CSFP em 25,75% dos pacientes. Os desfechos clínicos analisaram que a incidência de acidente vascular cerebral (AVC) foi significativamente maior no grupo CNP do que no grupo CSFP (6 (24%) vs. 6 (2,70%), p <0,001) e a de ECAM foi significativamente maior no grupo CNP do que no grupo CSFP (11 (44%) vs. 51 (23,10%), p = 0,022). A análise de regressão logística condicional forward demonstrou que o índice de massa corporal (IMC) (OR = 1,11, IC95%: 1,00-1,24, p = 0,038) e frequência cardíaca (FC) basal (OR = 0,923, IC 95%: 0,88-0,96, p <0,001) foram os preditores independentes de CNP no IAMSSST.

**Conclusões:**

Pacientes com CNP têm piores resultados clínicos e um maior risco de AVC em comparação com pacientes com CSFP no IAMSSST.

## Introdução

As síndromes coronárias agudas continuam sendo uma das principais causas de mortalidade e morbidade nos países industrializados e estão se tornando um problema cada vez mais importante nos países em desenvolvimento, apesar das melhoras em seu manejo e sua prevenção.^1^ Entre as síndromes coronárias agudas, os pacientes com infarto agudo do miocárdio sem supradesnivelamento do segmento ST (IAMSSST) apresentam os piores desfechos de longo prazo.^2^ Poucos estudos, entretanto, relataram os desfechos no IAMSSST, e esses relatos não esclareceram a diferença entre as características dos subgrupos fenômeno de *slow-flow* (CSFP, do inglês *coronary slow-flow phenomenon*) e fenômeno de *no-reflow* coronariano (CNP, do inglês *coronary no-reflow phenomenon*) na prática clínica, tanto no hospital quanto no seguimento de longo prazo, dentro de uma perspectiva do ‘mundo real’.^3^^,^^4^ Na ausência de doença arterial coronária obstrutiva, o fluxo coronário TIMI-II e a opacificação coronária tardia são definidos como CSFP.^5^ Além disso, o fluxo TIMI 0-I sem dissecção, obstrução mecânica, estenose residual significativa, espasmo ou trombo da artéria coronária são definidos como CNP angiográfico.^6^ Os mecanismos subjacentes no CNP e CSFP são inflamação, microembolização aterotrombótica, ativação de neutrófilos e plaquetária, que desencadeiam a liberação de radicais livres de oxigênio, enzimas proteolíticas e mediadores pró-inflamatórios, que podem causar dano tecidual e endotelial especialmente em miócitos com lesões graves.^5^^,^^6^

Além disso, não está claro sob que circunstâncias as diferenças nas características clínicas e nos desfechos persistem em pacientes com IAMSSST. Também não há evidências na literatura sobre como o fenômeno de *slow-flow* pode afetar os resultados no IAMSSST. Além disso, a comparação dos resultados entre CSFP e CNP em pacientes com IAMSSST não foi abordada na literatura. Nossa hipótese é que os piores desfechos clínicos no IAMSSST estão fortemente relacionados ao não-fluxo TIMI III nas artérias coronárias e especialmente no subconjunto do grupo CNP. No presente estudo, nosso objetivo foi investigar as características clínicas e comparar os principais desfechos cardiovasculares entre os grupos CSFP e CNP em pacientes com IAMSSST que foram seguidos por 12 meses.

## Métodos

**Figura 1:**
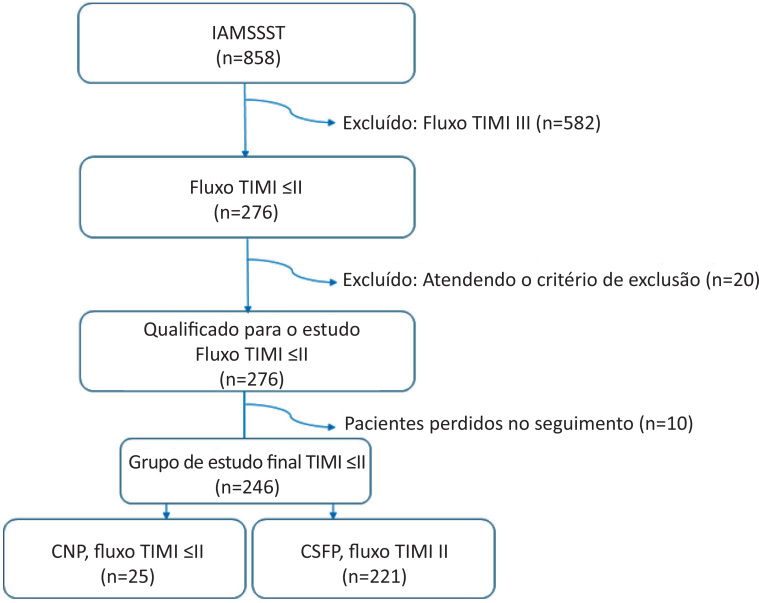
Diagrama mostrando a seleção dos grupos de estudo. IAMSSST: infarto agudo do miocárdio sem supradesnivelamento do segmento ST; TIMI: trombólise no infarto do miocárdio; CNP: fenômeno no-reflow coronariano; CSFP: fenômeno slow-flow coronariano.

Para este estudo de centro único, conduzido prospectivamente, um total de 858 pacientes com idade entre 18 e 90 anos foi incluído entre junho de 2016 e junho de 2018 no Bezmialem University Hospital, que foram diagnosticados com IAMSSST e submetidos a ICP precoce nas 24 horas desde o início dos sintomas (Figura1). Pacientes com fluxo TIMI III, cirurgia de revascularização do miocárdio (CABG), choque cardiogênico, edema pulmonar, sinais de disfunção ventricular esquerda aguda, trombose de stent, submetidos a aspiração de trombo no evento índice, com doença infecciosa ou neoplásica aguda ou crônica, doença renal moderada-grave e doença hepática crônica foram excluídos deste estudo (n = 602). De acordo com os resultados finais das características angiográficas do fluxo TIMI da artéria culpada tratada, um total de 25 pacientes com CNP comprovado por angiografia foram inscritos no grupo CNP, enquanto 221 pacientes com comprovação angiográfica de CSFP foram inscritos no grupo CSFP. Todos os pacientes receberam um total de 300 mg de ácido acetilsalicílico e uma dose de ataque (600 mg) de clopidogrel e heparina NF (100 mg/kg) durante a ICP. As informações de seguimento foram obtidas de registros hospitalares e após 1, 3, 6 e 12 meses durante as consultas feitas pelo paciente com o mesmo investigador. Os endpoints deste estudo foram obtidos a partir de prontuários hospitalares e atestados de óbito ou contato telefônico com parentes dos pacientes. Os eventos cardiovasculares adversos maiores (ECAM) foram definidos como mortalidade por todas as causas, morte cardiovascular, acidente vascular cerebral e reinfarto do miocárdio. Todos os participantes deram consentimento informado por escrito antes da participação no estudo e o estudo foi aprovado pelo comitê de ética local (Número: 7/70-04/17). Além disso, o estudo foi conduzido de acordo com os princípios da Declaração de Helsinque.

### Avaliação Bioquímica

Amostras de sangue venoso foram coletadas da veia antecubital imediatamente após a hospitalização antes da ICP. Um eletrocardiograma de 12 derivações foi obtido no momento da hospitalização no pronto-socorro e a frequência cardíaca (FC) foi registrada. A taxa de filtração glomerular estimada (TFGe) de cada paciente foi calculada utilizando-se a equação *Chronic Kidney Disease Epidemiology Collaboration*. O IMC foi calculado através da fórmula: peso (kg) /altura² (m²). A avaliação bioquímica de rotina do sangue, parâmetros lipídicos e níveis de troponina I cardíaca foram medidos em um autoanalisador padrão. O hemograma foi realizado com um autoanalisador Sysmex K-1000 (Block Scientific, Bohemia, NY, EUA). As amostras foram centrifugadas a 3000 rpm durante 10 min, e o sobrenadante e o soro foram separados das amostras, e em seguida, foram congelados a -80°C até análise posterior. A medida dos níveis de creatinina sérica foi repetida após 72 horas, depois da administração do meio de contraste (MC). A nefropatia induzida por contraste foi definida como um aumento absoluto de 0,5 mg/dL no nível de creatinina sérica acima da linha basal ou ≥25% de aumento relativo no nível de creatinina sérica basal nas 72 horas de exposição ao MC.

Diagnóstico de infarto do miocárdio sem elevação do segmento ST

O diagnóstico de IAMSSST foi feito na presença das seguintes características com base nas definições das diretrizes de prática clínica.^7^ Os pacientes com IAMSSST apresentaram dor ou desconforto torácico típico em repouso ou com esforço mínimo por pelo menos 10 minutos e o ECG inicial mostrou um ECG normal ou alterações isquêmicas, como infradesnivelamento do segmento ST ou inversão da onda T com nível elevado de troponina I cardíaca com pelo menos um valor acima do limite superior de referência do percentil 99.

### Fatores de Risco Cardiovascular

Após exames detalhados, o histórico médico de cada paciente foi coletado pelo mesmo investigador. Fatores de risco foram identificados para doença arterial coronariana (DAC), fatores de risco cardiovascular, incluindo idade, sexo, diabetes mellitus (DM), hipertensão (HT), hiperlipidemia (HPL) e tabagismo. Pacientes recebendo terapia anti-hipertensiva anterior ou com níveis de pressão arterial ≥140/90 mmHg em pelo menos duas medidas, foram considerados hipertensos.^8^ Pacientes previamente tratados com antidiabético oral e/ou terapia com insulina ou cuja glicemia de jejum era ≥ 125 mg/dL em pelo menos duas medidas foram considerados diabéticos.^9^ A presença de HPL foi considerada quando foi obtida uma medida de colesterol total >200 mg/dL ou de lipoproteína de baixa densidade-colesterol (LDL-C)>100 mg/dL, ou quando o paciente utilizava medicamento hipolipemiante, de acordo com a diretriz “Adult Treatment Panel III”.^10^ Pacientes que ainda utilizavam produtos do tabaco na admissão ao serviço de emergência e aqueles que tinham parado de fumar no último mês foram considerados fumantes.

### Ecocardiografia Transtorácica

Antes da alta, cada paciente foi submetido a um exame ecocardiográfico transtorácico utilizando um transdutor de 3,5 MHz (Vivid 7 GE Medical System, Horten, Noruega). Os exames e avaliações foram realizados de acordo com as recomendações das diretrizes da *American Echocardiography Unit*. O método de Simon foi utilizado para calcular a fração de ejeção do ventrículo esquerdo (FEVE).^11^

### Angiografia Coronária

Os procedimentos da angiografia coronária foram realizados por via femoral utilizando o sistema de angiografia Philips (Optimus 200 DCA e Integris Allura 9, Philips Medical Systems, Eindhoven, Países Baixos). A angiografia coronária e a ICP foram realizadas com meio de contraste não iônico, iso-osmolar (iodixanol, Visipaque 320mg/100mL, GE Healthcare, Cork, Irlanda) de acordo com as práticas clínicas padrão. A ICP da artéria relacionada ao infarto foi realizada. As imagens angiográficas foram obtidas a uma taxa de pelo menos 80 quadros e gravadas a uma taxa de 25 quadros por segundo. Pelo menos dois cardiologistas especialistas examinaram a anatomia coronária e o grau de fluxo TIMI *offline*. O fluxo TIMI das coronárias foi determinado pelo número quantitativo da contagem de quadros como descrito por Gibson et al.^12^ Fluxos TIMI de 0-I sem dissecção, obstrução mecânica, estenose residual significativa, espasmo ou trombo da artéria coronária foram definidos como CNP angiográfico. Na ausência de doença arterial coronária obstrutiva, o fluxo coronário TIMI-II e a opacificação coronária tardia foram definidos como CSFP. Os pacientes com CNP receberam tratamento com inibidores da glicoproteína IIb/IIIa intracoronários (IC) (inib. da Gp-IIb/IIIa) ou adenosina IC ou epinefrina IC. Após o procedimento, todos os pacientes receberam hidratação venosa (IV) com solução salina isotônica (1mL/kg/h) por pelo menos 12 horas.

### Análise Estatística

A análise dos dados foi realizada com o pacote de software estatístico SPSS versão 22.0 (SPSS Inc., Chicago, IL, EUA). A distribuição normal de uma variável contínua foi avaliada pelo teste de Kolmogorov-Smirnov. O teste *t* para amostras independentes ou o teste U de Mann-Whitney foram utilizados para comparar variáveis contínuas dependendo se os pressupostos estatísticos fossem cumpridos ou não. As variáveis contínuas foram expressas em média e desvio padrão se distribuídas normalmente, ou medianas e percentis 25 e 75 caso não satisfizessem a suposição de normalidade. As variáveis categóricas foram expressas em número (porcentagem). O teste de qui-quadrado foi utilizado para comparar variáveis categóricas. A correlação entre as variáveis foi realizada através da análise do teste de *log-rank* de Spearman. O método de Kaplan-Meier foi utilizado para estimar as taxas de sobrevida livre de eventos. A análise da curva característica de operação do receptor (ROC, do inglês *Receiver Operating Characteristic*) foi realizada para determinar o valor preditivo do IMC e da FC para o CNP. Foi realizada a análise de regressão logística univariada, e as variáveis que se mostraram estatisticamente significativas (p<0,1) foram avaliadas com a análise de regressão logística multivariada. O *odds ratio* e o intervalo de confiança de 95% de cada variável independente foram calculados. Um valor de P bicaudal <0,05 foi considerado significativo.

## Resultados

**Figura 2:**
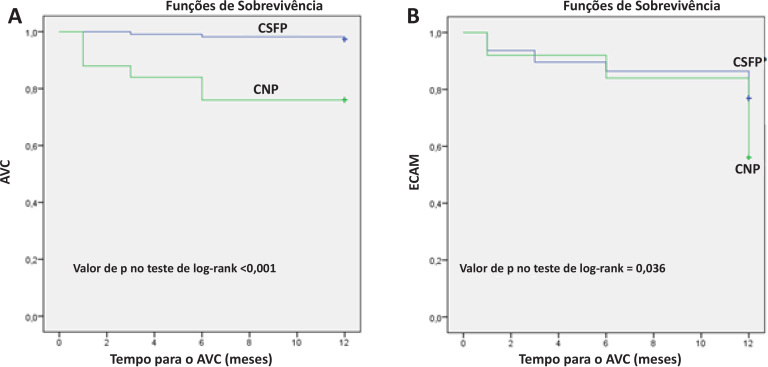
(A) Estimativas de Kaplan-Meier para AVC. (B) Estimativas de Kaplan-Meier para ECAM. AVC: acidente vascular cerebral; ECAM: eventos cardíacos adversos maiores; CNP: fenômeno no-reflow coronariano; CSFP: fenômeno slow-flow coronariano.

Neste estudo, incluímos um total de 858 pacientes com IAMSSST e, por fim, concluímos o presente estudo com 246 pacientes (171 homens; média de idade: 61,69 ± 12,60 anos). Em pacientes com IAMSSST, o CNP foi observado em 2,91% (n = 25) e o CSFP foi observado em 25,75% (n = 221). Para a população final do estudo, o grupo CNP teve 25 (10,16%) pacientes e o grupo CSFP, 221 (89,84%) pacientes. Os achados demográficos são descritos na Tabela 1. Além disso, a classe NYHA, a frequência cardíaca, o tempo de internação hospitalar, o escore de Mehran e a TFGe foram significativamente associados ao EuroSCORE-II (p<0,05) (Tabela 2). Os achados do seguimento clínico foram descritos na Tabela 3. Não identificamos nenhum acidente vascular cerebral (AVC) hemorrágico durante o seguimento. As estimativas de Kaplan-Meier para as taxas de AVC e ECAM são descritas na Figura 2A e Figura 2B. A análise de regressão logística condicional *forward* demonstrou que o IMC e a FC foram os preditores independentes de CNP (Tabela 4).

**Tabela 1:** Características basais e laboratoriais dos pacientes

Variável, n (%)	CNP, n=25 (10.16)	CSFP, n=221 (89.84)	p-valor
Idade, a	66,28±14,14	61,17±12,34	0,057
Sexo feminino, n (%)	12 (48)	63 (28,50)	0,045
IMC, kg / m²	30,51±3,99	28,34±4,55	0,015
HT, n (%)	19 (76)	129 (58,40)	0,088
DM, n (%)	10 (40)	70 (31,70)	0,400
HL, n (%)	9 (36)	95 (43)	0,503
Fumante, n (%)	15 (60)	132 (59,70)	0,979
Histórico familiar, n (%)	8 (32)	73 (33)	0,917
DAP, n (%)	5 (20)	13 (5,90)	0,010
DPOC, n (%)	5 (20)	31 (14)	0,423
FEVE,%	50±7,40	52,29±7,19	0,126
Glicose, mg/dL	115 (90,50-174)	106 (96-146)	0,719
Ácido úrico, mg/dL	5,60 (4,55-7,25)	5,80 (4,20-6,90)	0,303
Creatinina, mg/dL	0,86 (0,77-1,23)	0,87 (0,76-1,05)	0,175
TFGe, mL/min por 1,73 m²	70,90±25,95	82,86±20,80	0,021
Triglicérides, mg/dL	153 (125-195)	147 (110,5-180)	0,353
LDL, mg/dL	135 (114-171)	125 (98-149)	0,051
HTC,%	40,60 (35,80-42)	41 (37,10-43,15)	0,344
Plaquetas, 10³/uL	220 (185-266)	225 (190-276,50)	0,428
Pico de troponina-I, pg/mL	814 (156-5693,50)	146 (116-2113)	0,037
PCR-us, mg/dL	0,10 (0,01-0,57)	0,18 (0,04-0,50)	0,836
Frequência cardíaca, bpm	69,60±19,86	78,81±13,46	<0,001
Tempo de hospitalização, d.	3,40±0,95	3,00±0,88	0,015
Escore de Mehran	7,56±6,20	5,24±4,91	0,017
Desenvolvimento NIC, n (%)	4 (16)	19 (8,60)	0,228
Classe NYHA	2,48±0,50	2,04±0,40	<0,001
EuroSCORE II,%	3,96±3,95	2,14±2,32	<0,001
*Medicamentos*, n (%)			
Inibidores de ECA	17 (68)	110 (49,80)	0,084
BRA	7 (28)	75 (33,90)	0,551
Betabloqueador	24 (96)	212 (95,90)	0,986
BCC	9 (36)	52 (23,50)	0,171
Estatina	25 (100)	194 (87,80)	0,064
Nitrato	11 (44)	73 (33)	0,273
DAOs	10 (40)	68 (30,80)	0,347
Diuréticos	13 (52)	71 (32,10)	0,047
Gp-IIb / IIIa inh. IC	25 (100)	8 (3,61)	<0,001
Adenosina IC	25 (100)	1 (0,45)	<0,001
Epinefrina IC	25 (100)	1 (0,45)	<0,001

Os valores são expressos em média ± DP ou números e porcentagens ou mediana e percentis 25-75. Valor de p para dados categóricos do Qui-quadrado. Valor de p para o teste *t* de amostras independentes ou o teste U de Mann-Whitney foi utilizado para comparar variáveis contínuas. CNP: fenômeno no-reflow coronariano; CSFP: fenômeno slow-flow coronariano; a: anos; IMC: Índice de Massa Corporal; HT: hipertensão; DM: diabetes mellitus tipo 2; HL: hiperlipidemia; DAP: doença arterial periférica; DPOC: doença pulmonar obstrutiva crônica; FEVE: fração de ejeção do ventrículo esquerdo; TFGe: taxa de filtração glomerular estimada; LDL: lipoproteína de baixa densidade; HTC: hematócrito; PCR-us: proteína C reativa de alta sensibilidade; bpm: batimentos por minuto; d: dias; NIC: Nefropatia induzida por contraste; NYHA: Classificação Funcional da New York Heart Association; EuroSCORE: European System for Cardiac Operative Risk Evaluation; Inibidores ECA: inibidores da enzima de conversão da angiotensina; BRA: bloqueadores do receptor da angiotensina; B bloqueador: betabloqueador; BCC. bloqueadores dos canais de cálcio; DAOs: drogas anti-hiperglicêmicas orais; IC: intracoronário; Inibidores Gp-IIb / IIIa: inibidores da glicoproteína-IIb/IIIa.

**Tabela 2:** Características basais significativamente associadas ao EuroSCORE II

Variável	r	p-valor
Classe NYHA	0,590	<0,001
Frequência cardíaca	0,192	0,003
Tempo de hospitalização	0,468	<0,001
Escore de Mehran	0,763	<0,001
TFGe	-0,671	<0,001

EuroSCORE II: European System for Cardiac Operative Risk Evaluation II; r: coeficiente de correlação de log-rank de Spearman, NYHA: Classificação Funcional da New York Heart Association; TFGe: taxa de filtração glomerular estimada.

**Tabela 3:** Achados do seguimento clínico em um ano

Variável, n (%)	CNP, n=25 (10.16)	CSFP, n=221 (89.84)	p-valor
Mortalidade por todas as causas	4 (16)	29 (13,10)	0,689
Morte Cardiovascular	4 (16)	23 (10,40)	0,396
AVC	6 (24)	6 (2,70)	<0,001
Reinfarto do miocárdio	3 (12)	25 (11,30)	0,918
ECAM	11 (44)	51 (23,10)	0,022

Os valores são expressos como números e porcentagens. CNP: fenômeno no-reflow coronariano; CSFP: fenômeno slow-flow coronariano; AVC: acidente vascular cerebral; ECAM: Eventos cardiovasculares adversos maiores.

**Tabela 4:** Preditores independentes de CNP

Variável	OR	IC95%	p-valor
IMC	1,11	1,00-1,24	0,038
FC	0,923	0,88-0,96	<0,001

OR: Odds ratio; IC: intervalo de confiança; IMC: índice de massa corporal; FC: frequência cardíaca.

**Figura 3:**
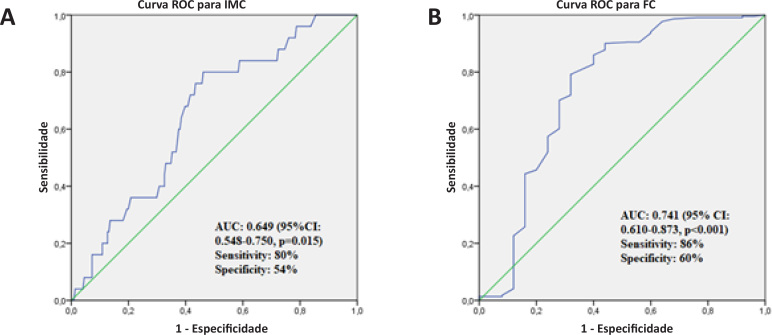
(A) Curva ROC para a especificidade e sensibilidade do IMC. (B) Curva ROC para a especificidade e sensibilidade da FC. IMC: índice de massa corporal; FC: frequência cardíaca; ROC: Curva da característica de operação do receptor; AUC: área sob a curva; IC: intervalo de confiança.

Na análise ROC, um IMC >28,38 kg/m^2^ previu a presença de CNP com 80% de sensibilidade e 54% de especificidade. A área sob a curva foi de 0,649 (IC 95%: 0,548–0,750, p = 0,015) (Figura 3A). Além disso, a FC < 66,5 bpm previu a presença de CNP com 86% de sensibilidade e 60% de especificidade. A área sob a curva foi de 0,741 (IC 95%: 0,88-0,96, p <0,001) (Figura 3B).

## Discussão

O principal achado desta pesquisa foi que os dois determinantes do CNP em pacientes com IAMSSST foram os níveis aumentados de IMC e FC mais baixa. Além disso, em pacientes com IAMSSST, o CNP foi significativamente associado a desfechos ruins. Mostramos que os valores de IMC > 28,38 kg/m^2^ sugerem a presença de CNP no IAMSSST. Além disso, a FC < 66,5 bpm sugere a presença de CNP no IAMSSST. Que seja de nosso conhecimento, este é o primeiro relato na literatura que demonstra a relação entre o IMC e FC mais baixa em pacientes com CNP com IAMSSST. Em nosso estudo, os resultados do seguimento clínico de um ano mostraram que as incidências de AVC e ECAM foram significativamente maiores no grupo CNP. Neste estudo, mostramos que o CNP piorou os desfechos dos pacientes com IAMSSST.

CSFP e CNP não são achados frequentes, com uma incidência de aproximadamente 1% em pacientes submetidos à angiografia coronariana; entretanto, em relação aos dados publicados, as frequências estimadas de CNP e CSFP variam de 1% a 60% na síndrome coronariana aguda.^13^^,^^14^ Neste estudo, o CNP foi observado em 2,91% e o CSFP em 25,75% da população estudada. CSFP e CNP estão associados a desfechos clínicos ruins em curto e longo prazo.^15^ Em particular, o CNP é um preditor significativo de desfechos cardíacos ruins no IAMSSST.^13^^,^^16^ Consistente com os dados publicados, encontramos os piores desfechos no grupo CNP. Em nosso estudo, os achados do seguimento clínico de um ano demonstraram que os desfechos de ECAM e AVC foram significativamente maiores no grupo CNP. No grupo CNP, a probabilidade de AVC foi 8,88 vezes maior do que no grupo CSFP.

Além disso, no grupo CNP, observamos uma probabilidade de ECAM 1,90 vezes maior do que no grupo PFC. Meta-análises anteriores, incluindo estudos retrospectivos e prospectivos, encontraram uma associação positiva entre troponina cardíaca e eventos adversos no IAMSSST.^17^ Neste estudo, consistente com a literatura, encontramos um nível de pico de troponina-I significativamente mais alto no grupo CNP. Enquanto isso, o acidente vascular cerebral foi associado à carga de um trombo. De acordo com nossa pesquisa, o mecanismo relacionado que causa esse evento adverso é a ativação contínua do trombo após o evento índice, e consideramos que essa pode ser a principal razão para o aumento do risco de acidente vascular cerebral. Embora todos os pacientes com IAMSSST tenham sido tratados regularmente com drogas antitrombóticas, o AVC ocorreu com uma incidência significativamente mais alta no grupo CNP. Assim, após a alta, tais pacientes precisam ser cuidadosamente monitorados. Além disso, o IMC é o método mais comumente utilizado para avaliação de risco cardiovascular e obesidade.^18^

Em pacientes com IAMSSST, Bakirci et al.^19^ descobriram que a gordura epicárdica, que está aumentada em pacientes obesos, está associada ao fluxo coronariano prejudicado.^19^ Estudos recentes sugeriram que o CNP é mais comumente visto em combinação com hiperglicemia, hipercolesterolemia e insuficiência renal leve a moderada.^20^ No presente estudo, encontramos níveis de TFGe significativamente mais baixas e escores de Mehran mais altos no grupo CNP, consistentes com a literatura. Além disso, em nosso estudo, os pacientes do grupo CNP apresentaram IMC significativamente maior e consideramos que isso pode estar associado a um risco aumentado de acidente vascular cerebral. Portanto, o cálculo do IMC pode ser um método útil para estimar os desfechos cardíacos em pacientes com IAMSSST e CNP. Também consideramos que a diminuição do IMC pode proteger os pacientes contra um AVC.

Enquanto isso, estudos randomizados mostraram que o uso de cateter de aspiração manual de trombo pode fornecer melhor perfusão microvascular e resultados em longo prazo em comparação com pacientes controle.^21^ Entretanto, o uso de aspiração de trombo pode causar acidente vascular cerebral devido a complicações do dispositivo, razão pela qual em nosso estudo excluímos os pacientes (n = 6) submetidos a aspiração de trombo durante o procedimento índice, de forma que não afetasse o endpoint de AVC. O uso rotineiro de inibidores de plaquetas (inibidores da Gp-IIb / IIIa., Abciximab, tirofiban), nicorandil, nitroprussiato e adenosina mostraram efeitos benéficos na perfusão miocárdica no IAMSSST.^22^ Além disso, Aksu et al. descobriram que o uso de epinefrina intracoronária teve um efeito benéfico no CNP.^23^ Além disso, Skelding et al.^24^ descobriram que um aumento da pressão arterial na circulação coronariana e taquicardia podem ser outros efeitos benéficos potenciais da epinefrina.^24^ Em nosso estudo, consistente com a literatura, descobrimos que a FC mais baixa foi independentemente associada com o CNP em pacientes com IAMSSST. Se a microcirculação for lenta, o CNP ocorre, e sugerimos que a FC mais baixa poderia ser um indicador de CNP em pacientes com IAMSSST. Os médicos intervencionistas devem estar cientes da FC do paciente, e o paciente com menor FC deve ser considerado como candidato a CNP antes de iniciar a ICP. Apesar dos resultados encorajadores de nosso estudo, os achados de FC mais baixa devem ser explicados por estudos grandes e randomizados.

### Limitações

Em primeiro lugar, embora um modelo multivariado tenha sido utilizado para ajustar as variáveis de confusão, um viés era inevitável, uma vez que sesta foi uma análise unicêntrica com um tamanho de amostra razoavelmente pequeno. Ensaios multicêntricos com mais pacientes podem mostrar melhores resultados e fornecer mais dados. Em segundo lugar, apenas parâmetros angiográficos foram usados na determinação do CNP e CSFP; a microcirculação não foi avaliada diretamente; por outro lado, nem a ecocardiografia, nem os pacientes foram avaliados com ressonância magnética (RM) para confirmar a reperfusão microvascular adequada. A RM é o melhor método para avaliação da obstrução microvascular. Terceiro, para avaliar os resultados clínicos em longo prazo, um período de seguimento de um ano pode não ser adequado. Esses fatores limitam nosso estudo.

## Conclusão

Os dois determinantes do CNP em pacientes com IAMSSST foram níveis aumentados de IMC e FC mais baixa. Em nosso estudo, os resultados do seguimento clínico de um ano mostraram que a incidência de AVC e ECAM foi significativamente mais elevada no grupo CNP. Este estudo mostrou que o CNP piorou os desfechos dos pacientes com IAMSSST.
