# Supplementary material for: Comparação dos Resultados entre os Fenômenos de No-Reflow e Slow-Flow Coronariano em Pacientes sem IAMSSST
Source: Arq Bras Cardiol. 2021 May 6;116(5):856–64. [Article in Portuguese] doi: 10.36660/abc.20190905 (PMC8121461; doi:10.36660/abc.20190905)
Supplement: Translation

# Translation: Comparison of the Outcomes between Coronary No-Reflow and Slow-Flow Phenomenon in Non-STEMI Patients

**Authors:** Mustafa Ahmet Huyut

**Affiliations:** 1 Istanbul Turkey Yeni Yuzyil University, Faculty of Medicine, Department of Cardiology, Istambul – Turkey.

**Keywords:** Myocardial Infarction, No-Reflow Phenomenon, Percutaneous Coronary Interventon, Acute Coronary Syndrome/complications, Risk Factors, Coronary Angiography, Stroke

## Abstract

**Background:**

Coronary slow-flow phenomenon (CSFP) and coronary no-reflow phenomenon (CNP) are associated with increased risk of major cardiovascular adverse events (MACE).

**Objectives:**

This study aimed to evaluate and compare the one-year clinical follow-up outcomes among patients with CNP and CSFP who underwent percutaneous coronary interventions (PCI) in non-ST elevation myocardial infarction (NSTEMI).

**Methods:**

This study included a total of 858 patients who were diagnosed with NSTEMI and underwent PCI within 24 h of symptom onset. The patients were divided into two groups, the CSFP group (n=221) and the CNP group (n=25), regarding the angiographic characteristics of thrombolysis in myocardial infarction (TIMI) flow of the infarct-related artery. Patients were followed for one-year. A p-value of <0.05 was considered significant.

**Results:**

CNP was observed in 2.91%, and CSFP was observed in 25.75% of the patients. Clinical endpoints analyzed that stroke was significantly higher in the CNP group than in the CSFP group (6 (24%) vs. 6 (2.70%), p<0.001) and MACE was significantly higher in the CNP group than in the CSFP group (11 (44%) vs. 51 (23.10%), p=0.022). Forward conditional logistic regression analysis demonstrated that body mass index (BMI) (OR=1.11, 95%CI: 1.00-1.24, p=0.038) and baseline heart rate (HR) (OR=0.923, 95%CI: 0.88-0.96, p<0.001) were the independent predictors of CNP in NSTEMI.

**Conclusion:**

CNP patients have worse clinical outcomes and a higher risk of stroke compared with CSFP patients in NSTEMI. (Arq Bras Cardiol. 2021; 116(5):856-864)

## Introduction

Acute coronary syndromes remain a major cause of mortality and morbidity in industrialized countries and are becoming an increasingly important problem in developing countries, despite improvements in its management and prevention.^1^ Among the acute coronary syndromes, patients with non-ST elevation myocardial infarction (NSTEMI) have been shown to have worse long-term outcomes.^2^ Few studies have, however, reported on the outcomes in NSTEMI, but these reports have not clarified the difference between coronary slow-flow phenomenon (CSFP) and coronary no-reflow phenomenon (CNP) subgroups’ characteristics in clinical practice, both in the hospital and over the long term follow-up, from a ‘real-world’ perspective.^3^^,^^4^ In the absence of obstructive coronary artery disease, TIMI-II coronary flow and delayed coronary opacification are defined as CSFP.^5^ In addition, TIMI 0-I flows without dissection, mechanical obstruction, significant residual stenosis, spasm or coronary artery thrombus are defined as angiographic CNP.^6^ The underlying mechanisms in CNP and CSFP are inflammation, atherothrombotic microembolization, neutrophil and platelet activation, which triggers the release of oxygen-free radicals, proteolytic enzymes, and proinflammatory mediators that can trigger tissue and endothelial damage, especially in critically-injured myocytes.^5^^,^^6^

Moreover, it is unclear under what circumstances the differences in clinical characteristics and outcomes persist in NSTEMI patients. Also, there is no evidence in the literature about how slow-flow could affect the outcomes in NSTEMI. Additionally, the comparison of outcomes between CSFP and CNP in NSTEMI patients has not been addressed in the literature. We hypothesized that the worst clinical outcomes in NSTEMI are strongly related to the non-TIMI III flow in the coronary arteries and especially in the CNP group subset. In the present study, we aimed to investigate the clinical characteristics and compare the major cardiovascular outcomes between CSFP and CNP in NSTEMI patients who were followed for 12 months.

## Methods

For this single-center, prospectively conducted study, 858 patients aged between 18 and 90 years were enrolled between June 2016 and June 2018 at Bezmialem University Hospital, who were diagnosed with NSTEMI and submitted to early PCI within 24 hours of symptom onset (Figure1). Patients with TIMI III flow, coronary artery bypass graft (CABG), cardiogenic shock, pulmonary edema, signs of acute left ventricular dysfunction, stent thrombosis, underwent thrombus aspiration in index event, had acute or chronic infective or neoplastic disease, moderate-to-severe chronic kidney disease, and chronic liver disease were excluded from this study (n=602). The final results of the angiographic characteristics of TIMI flow of the treated culprit artery assigned a total of 25 patients with angiographically-proven CNP to the CNP group and 221 patients with angiographically-proven CSFP to the CSFP group. All patients received a total of 300 mg acetylsalicylic acid and a loading dose (600 mg) of clopidogrel and UF heparin (100mg / kg) during the PCI. The follow-up information was obtained from hospital records and after 1, 3, 6, and 12 months during patients’ visits by the same investigator. The endpoints of this study were obtained from hospital records and death certificates, or telephone contact with the patients’ relatives. Major cardiovascular adverse events (MACE) was defined as all-cause mortality, cardiovascular death, stroke, and myocardial re-infarction. All participants gave written informed consent prior to study participation and the study was approved by the local ethics committee (Number:7/70-04/17). Furthermore, the study was conducted according to the provisions of the Declaration of Helsinki.

**Figure 1:** Diagram shows the selection of the study groups. Non-STEMI: non-ST elevation myocardial infarction; TIMI: thrombolysis in myocardial infarction; CNP: coronary no-reflow phenomenon; CSFP: coronary slow-flow phenomenon.

### Biochemical Assessment

Venous blood samples were taken from the antecubital vein immediately after admission to the hospital before PCI. A 12-lead electrocardiogram was obtained at the time of admission to the emergency department and heart rate (HR) was noted. The estimated glomerular filtration rate (eGFR) of each patient was calculated using the Chronic Kidney Disease Epidemiology Collaboration equation. The BMI was calculated using the formula weight (kg)/ height² (m²).Routine blood chemistry, lipid parameters, and cardiac troponin-I levels were measured using a standard auto-analyzer. Blood counts were measured in a Sysmex K-1000 (Block Scientific, Bohemia, NY, USA) auto-analyzer. Samples were centrifuged at 3000 rpm for 10 min, the supernatant and serum were separated in the samples and then they were frozen at -80° C until further analysis. The serum creatinine level measurement was repeated at 72 hours after contrast medium (CM) administration. Contrast-induced nephropathy was defined as a 0.5 mg/dL absolute increase in serum creatinine level above baseline or ≥25% relative increase in basal serum creatinine level within 72 hours of CM exposure.

### Diagnosis of non-ST-segment Elevation Myocardial Infarction

The diagnosis of NSTEMI was made in the presence of characteristics based on definitions from clinical practice guidelines.^7^ The NSTEMI patients had typical chest pain or discomfort occurring at rest or minimal exertion, for at least 10 minutes, and the initial ECG showed normal ECG or ischemic changes, such as ST-depression or T-wave inversion with elevated cardiac troponin-I level with at least 1 value above the 99^th^ percentile of the upper reference limit.

### Cardiovascular Risk Factors

After detailed examinations, the medical history of each patient was collected by the same investigator. Risk factors were identified for coronary artery disease (CAD), cardiovascular risk factors including age, gender, diabetes mellitus (DM), hypertension (HT), hyperlipidemia (HPL), and smoking status. Patients receiving prior antihypertensive therapy or blood pressure levels ≥140/90 mmHg, measured at least twice, were considered hypertensive.^8^ Patients previously treated with oral antidiabetic and/or insulin therapy or whose fasting blood glucose was as high as ≥125 mg/dL, after being measured at least twice, were considered diabetic.^9^ The presence of HPL was considered when a measurement of total cholesterol > 200 mg/dL or low-density lipoprotein cholesterol (LDL-C) >100 mg / dL was obtained, or when the patient used lipid-lowering medication in accordance with the “Adult Treatment Panel III” guideline.^10^ Patients who still used tobacco products on admission to the emergency service and those who had stopped smoking in the past month were considered smokers.

### Transthoracic Echocardiography

Before discharge, each patient underwent a transthoracic echocardiographic examination using a 3.5-MHz transducer (Vivid 7 GE Medical System, Horten, Norway). Examinations and assessments were carried out according to the recommendations of the American Echocardiography Unit guidelines. Simpson’s method was used to calculate left ventricular ejection fraction (LVEF).^11^

### Coronary Angiography

Coronary angiography procedures were performed via the femoral approach using a Philips (Optimus 200 DCA and Integris Allura 9, Philips Medical Systems, Eindhoven, Netherlands) angiography system. Coronary angiography and PCI were conducted using nonionic, iso-osmolar contrast media (iodixanol, Visipaque 320mg/100mL, GE Healthcare, Cork, Ireland) according to standard clinical practices. The PCI of the infarct-related artery was performed. Angiographic images were taken at a rate of at least 80 frames and recorded at a rate of 25 frames per second. At least two expert cardiologists evaluated coronary anatomy and TIMI flow grade offline. Coronary artery TIMI flow was determined by the quantitative number of frame counts as described by Gibson et al.^12^ TIMI 0-I flows without dissection, mechanical obstruction, significant residual stenosis, spasm or coronary artery thrombus were defined as angiographic CNP. In the absence of obstructive coronary artery disease, TIMI-II coronary flow and delayed coronary opacification are defined as CSFP. CNP patients received treatment with intracoronary (IC) glycoprotein IIb/IIIa inhibitors (Gp-IIb/IIIa inh.) or IC adenosine or IC epinephrine. After the procedure, all patients received intravenous (IV) hydration with isotonic saline (1mL/kg/h) for at least 12 hours.

### Statistical Analysis

Data analyses were performed using SPSS version 22.0 statistical software package (SPSS Inc., Chicago, IL, USA). The normal distribution of a continuous variable was assessed using the Kolmogorov-Smirnov test. The independent samples t-test or the Mann-Whitney U test was used to compare continuous variables depending on whether statistical assumptions were met or not. Continuous variables were expressed as mean and standard deviation if normally distributed, or medians and 25^th^ and 75^th^ percentiles if they did not satisfy the normal assumption. Categorical variables were expressed as number (percentage). The Chi-square test was used to compare categorical variables. The correlation between variables was performed using Spearman’s rank-order correlation analysis. The Kaplan-Meier method was used to estimate event-free survival rates. Receiver operating characteristic (ROC) curve analysis was performed to determine the BMI and the HR predictive value for CNP. Univariate logistic regression analysis was performed, and the variables that were found to be statistically significant (p<0.1) were analyzed with multivariate logistic regression analysis. The odds ratio and 95% confidence interval of each independent variable were calculated. A two-tailed p value of <0.05 was considered significant.

## Results

In this study, we included a total of 858 NSTEMI patients and at the end, we concluded the present study with 246 patients (171 males; mean age: 61.69±12.60 years). In NSTEMI patients, CNP was observed in 2.91% (n=25) and CSFP was observed in 25.75% (n=221). Regarding the final study population, the CNP group had 25 (10.16%) patients and the CSFP group had 221 (89.84%) patients. Demographic findings are described in Table1. Moreover, NYHA class, heart rate, hospital length of stay, Mehran score, and eGFR were significantly associated with EuroSCORE-II (p<0.05) (Table 2). Clinical follow-up findings were described in Table 3. We did not identify any hemorrhagic stroke during follow-up. Kaplan-Meier estimates for stroke and MACE rates are described in Figure 2A and Figure 2B. Forward conditional logistic regression analysis demonstrated that BMI and HR were the independent predictors of CNP (Table 4).

**Table 1:** Baseline and laboratory characteristics of the patients

Variable, n (%)	CNP, n=25 (10.16)	CSFP, n=221 (89.84)	p-value
Age, y	66.28±14.14	61.17±12.34	0.057
Female gender, n (%)	12 (48)	63 (28.50)	0.045
BMI, kg/m²	30.51±3.99	28.34±4.55	0.015
HT, n (%)	19 (76)	129 (58.40)	0.088
DM, n (%)	10 (40)	70 (31.70)	0.400
HL, n (%)	9 (36)	95 (43)	0.503
Smoker, n (%)	15 (60)	132 (59.70)	0.979
Family History, n (%)	8 (32)	73 (33)	0.917
PAD, n (%)	5 (20)	13 (5.90)	0.010
COPD, n (%)	5 (20)	31 (14)	0.423
LVEF, %	50±7.40	52.29±7.19	0.126
Glucose, mg/dl	115 (90.50-174)	106 (96-146)	0.719
Uric acid, mg/dl	5.60 (4.55-7.25)	5.80 (4.20-6.90)	0.303
Creatinine, mg/dl	0.86 (0.77-1.23)	0.87 (0.76-1.05)	0.175
eGFR, mL/min per 1.73 m²	70.90±25.95	82.86±20.80	0.021
Triglycerides, mg/dL	153 (125-195)	147 (110.5-180)	0.353
LDL, mg/dL	135 (114-171)	125 (98-149)	0.051
HTC, %	40.60 (35.80-42)	41 (37.10-43.15)	0.344
Platelets, 10³/uL	220 (185-266)	225 (190-276.50)	0.428
Peak Troponin-I, pg/ml	814 (156-5693.50)	146 (116-2113)	0.037
hs-CRP, mg/dL	0.10 (0.01-0.57)	0.18 (0.04-0.50)	0.836
Heart Rate, bpm	69.60±19.86	78.81±13.46	<0.001
Hospital length of stay, d.	3.40±0.95	3.00±0.88	0.015
Mehran Score	7.56±6.20	5.24±4.91	0.017
CIN development, n (%)	4 (16)	19 (8.60)	0.228
NYHA class	2.48±0.50	2.04±0.40	<0.001
EuroSCORE II, %	3.96±3.95	2.14±2.32	<0.001
*Medications, n (%)*			
Ace inh	17 (68)	110 (49.80)	0.084
ARB	7 (28)	75 (33.90)	0.551
B-blocker	24 (96)	212 (95.90)	0.986
CCB	9 (36)	52 (23.50)	0.171
Statin	25 (100)	194 (87.80)	0.064
Nitrate	11 (44)	73 (33)	0.273
OAD	10 (40)	68 (30.80)	0.347
Diuretic	13 (52)	71 (32.10)	0.047
IC Gp-IIb/IIIa inh.	25 (100)	8 (3.61)	<0.001
IC adenosine	25 (100)	1 (0.45)	<0.001
IC epinephrine	25 (100)	1 (0.45)	<0.001

Values are mean±SD or numbers and percentages or median and 25th-75th percentiles. The p-value for categorical data from Chi-square. The p-value for independent samples t-test or the Mann-Whitney U test was used to compare continuous variables. CNP: coronary no-reflow phenomenon; CSFP: coronary slow-flow phenomenon; Y: year; BMI: body mass index; HT: hypertension; DM: diabetes mellitus type 2; HL: hyperlipidemia; PAD: peripheral arterial disease; COPD: chronic obstructive pulmonary disease; LVEF: left ventricular ejection fraction; eGFR: estimated glomerular filtration rate; LDL: low-density lipoprotein; HTC: hematocrit; hs-CRP: high-sensitivity C-reactive protein; bpm: beats per minute; d: days; CIN: Contrast-induced nephropathy; NYHA: the New York Heart Association Functional Classification; EuroSCORE: European System for Cardiac Operative Risk Evaluation; ACE inh: angiotensin-converting enzyme inhibitors; ARB: angiotensin receptor blockers; B-blocker: beta-blocker; CCB. calcium channel blockers; OAD: oral antihyperglycemic drugs; IC: intracoronary; Gp-IIb/IIIa inh: glycoprotein-IIb/IIIa inhib>

**Table 2:** Baseline characteristics significantly associated with EuroSCORE II

Variable	r	p-value
NYHA class	0.590	<0.001
Heart Rate	0.192	0.003
Hospital length of stay	0.468	<0.001
Mehran Score	0.763	<0.001
eGFR	-0.671	<0.001

EuroSCORE II: European System for Cardiac Operative Risk Evaluation II; r: Spearman’s rank correlation coefficient, NYHA: the New York Heart Association Functional Classification; eGFR: estimated glomerular filtration rate.

**Table 3:** One-year clinical follow-up findings

Variable, n (%)	CNP, n=25 (10.16)	CSFP, n=221 (89.84)	p-value
All-Cause Mortality	4 (16)	29 (13.10)	0.689
Cardiovascular Death	4 (16)	23 (10.40)	0.396
Stroke	6 (24)	6 (2.70)	<0.001
Myocardial re-infarction	3 (12)	25 (11.30)	0.918
MACE	11 (44)	51 (23.10)	0.022

Values are numbers and percentages. CNP: coronary no-reflow phenomenon; CSFP: coronary slow-flow phenomenon; MACE: Major Adverse cardiovascular events.

**Figure 2:** (A) Kaplan-Meier estimates for Stroke. (B) Kaplan-Meier estimates for MACE. MACE: major adverse cardiac events; CNP: coronary no-reflow phenomenon; CSFP: coronary slow-flow phenomenon.

**Table 4:** Independent predictors of CNP

Variable	OR	95% CI	p-value
BMI	1.11	1.00-1.24	0.038
HR	0.923	0.88-0.96	<0.001

OR: Odds ratio; CI: Confidence interval; BMI: body mass index; HR: heart rate.

In the ROC analysis, a BMI > 28.38 kg/m^2^ predicted the presence of CNP with 80% of sensitivity and 54% of specificity. The area under the curve was 0.649 (95%CI: 0.548–0.750, p=0.015) (Figure 3A). Moreover, HR < 66.5 bpm predicted the presence of CNP with 86% of sensitivity and 60% of specificity. The area under the curve was 0.741 (95%CI: 0.88–0.96, p<0.001) (Figure 3B).

**Figure 3:** (A) ROC curve for the specificity and sensitivity of BMI. (B) ROC curve for the specificity and sensitivity of HR. BMI: body mass index; HR: heart rate; ROC: receiver operating characteristic curve; AUC: area under the curve; CI: confidence interval.

## Discussion

The key finding of this research was that the two determinants of CNP in NSTEMI patients were increased BMI levels and lower HR. Additionally, in patients with NSTEMI, CNP was significantly associated with poor outcomes. We showed that BMI values > 28.38 kg/m^2^ suggest the presence of CNP in NSTEMI. Moreover, HR < 66.5 bpm suggests the presence of CNP in NSTEMI. To the best of our knowledge, this is the first report in the literature demonstrating the relationship between BMI and lower HR in CNP patients with NSTEMI. In our study, the results of the one-year clinical follow-up showed that the incidence of stroke and MACE was significantly higher in the CNP group. In this study, we showed that CNP worsened NSTEMI patients’ outcomes.

CSFP and CNP are not frequent findings, with an incidence of approximately 1% in patients undergoing coronary angiography; however, according to the published data, the estimated frequency of CNP and CSFP range from 1% to 60% in acute coronary syndrome.^13^^,^^14^ In this study, CNP was observed in 2.91% and CSFP was observed in 25.75% of the study population. CSFP and CNP are associated with poor short-term and long-term clinical outcomes.^15^ In particular, CNP is a significant predictor of poor cardiac outcomes in NSTEMI.^13^^,^^16^ Consistent with the published data, we found the worst outcomes in the CNP group. In our study, the one-year clinical follow-up findings demonstrated that MACE and stroke outcomes were significantly higher in the CNP group. In the CNP group, the probability of stroke was 8.88-fold higher than in the CSFP group.

Moreover, in the CNP group, we observed that the probability of MACE was 1.90-fold higher than in the CSFP group. Previous meta-analyses including both retrospective and prospective studies found a positive association between cardiac troponin and adverse events in NSTEMI.^17^ In this study, consistent with the literature, we found a significantly higher peak troponin-I level in the CNP group. Meanwhile, stroke was associated with thrombus burden. According to our research, the associated mechanism causing this adverse event is continuing thrombus activation after the index event, and we considered that may be the main reason for the increased risk of stroke. Although all NSTEMI patients were regularly treated with antithrombotic drugs, stoke occurred with a significantly higher incidence in the CNP group. Thus, after discharge, such patients should be carefully monitored. In addition, BMI is the most commonly used method for cardiovascular risk and obesity assessment.^18^

In patients with NSTEMI, Bakirci et al.^19^ found that epicardial fat, which is increased in obese patients, is associated with an impaired coronary flow.^19^ Recent studies have suggested that CNP is more commonly seen in combination with hyperglycemia, hypercholesterolemia, and mild to moderate renal insufficiency.^20^ In the present study, we found significantly lower rates of eGFR and higher Mehran scores in the CNP group, consistent with the literature. Moreover, in our study, the CNP group patients had significantly higher BMI and we considered this might be associated with an increased risk of stroke. Therefore, the calculation of the BMI may be a useful method for estimating cardiac outcomes in NSTEMI patients with CNP. We also considered that decreasing BMI may protect patients from stroke.

Meanwhile, randomized studies showed that using a manual thrombus aspiration catheter may provide better microvascular perfusion and long-term outcomes when compared to control patients.^21^ However, the use of thrombus aspiration may cause stroke due to device complications, which is why in our study we excluded the patients (n=6) submitted to thrombus aspiration catheter during the index procedure, so it would not influence the stroke endpoint. The routine use of platelet inhibitors (Gp-IIb/IIIa inh., abciximab, tirofiban), nicorandil, nitroprusside, and adenosine have shown beneficial effects on myocardial perfusion in NSTEMI.^22^ In addition, Aksu et al. found that intracoronary epinephrine use had a beneficial effect on CNP.^23^ Moreover, Skelding et al.^24^ have found that increasing blood pressure in the coronary circulation and tachycardia may be other potential beneficial effects of epinephrine.^24^ In our study, consistent with the literature, we have found that a lower HR was independently associated with CNP in NSTEMI patients. If microcirculation is slow, CNP occurs, and we suggest that lower HR could be a CNP indicator in NSTEMI patients. Operators must be aware of the patient’s HR, and a patient with lower HR should be considered as a CNP candidate, before starting the PCI. In spite of the encouraging results of our study, the lower HR findings should be explained by large and randomized trials.

### Limitations

First, although a multivariate model was used to adjust confounding variables, a bias was inevitable, since this was a single-center analysis with a fairly small sample size. Multicenter trials with more patients might show better results and yield more data. Second, only angiographic parameters were used to determine CNP and CSFP; microcirculation was not directly evaluated; on the other hand, neither the echocardiography nor the patients were evaluated with magnetic resonance imaging (MRI) to confirm appropriate microvascular reperfusion. MRI is the best method for the evaluation of microvascular obstruction. Third, in order to assess long-term clinical results, a follow-up period of one year may not be adequate. These factors limit our study.

## Conclusion

The two determinants of CNP in NSTEMI patients were increased BMI and lower HR. In our study, the results of the one-year clinical follow-up showed that the incidence of stroke and MACE were significantly higher in the CNP group. This study showed that CNP worsened NSTEMI patients’ outcomes.
